# Casemix innovation: shifting to integrated care

**DOI:** 10.1186/1472-6963-11-S1-A24

**Published:** 2011-10-19

**Authors:** J Hofdijk

**Affiliations:** 1 Casemix, Utrecht, 3515 GA, The Netherlands

## Introduction

The vested interests of both hospitals and medical specialists in the current system are blocking a breakthrough to a real patient-centred approach within healthcare. Adapting the funding system to cut across traditional silos of healthcare seems to be the key; however, these vested interests are preventing the introduction of a process of change which is needed for this paramount innovation.

This national approach will be presented to assess its international potential, since all countries face the same long-term care crisis of a lack of resources to meet the health needs of their populations. It will also be presented as an innovative approach to Casemix, as the clustering of patients is done based on clinical dimensions, and not on groups defined by statistical analysis of available data.

## Methods

Since the year 2000, Dutch hospitals have begun to register data by episode, starting with a referral to a medical specialist at a hospital. At that moment, within the IT systems, a care-trajectory record is created for the specific health issue. The information at the level of the episode is used to support the physician during the care process, but it is also gathered into management databases at the institutional level. With this information, profiles can be created at different levels of aggregation, for example, for individual patients, at the provider level, at the level of diseases treated, and for many other ad hoc views.

By the structural linking of the data to the health issue of the patient, described by both care request and diagnosis, a new dimension has been created in the resource management of hospitals. Since the shift in the funding of hospitals from budgeting to contracting will be completed in 2012, hospitals need to change their information management strategies. In the presentation, examples of this newly developed management information will be presented.

The next step in the process of health reform dealt with chronic diseases. This was partially driven by the spectacular growth expectations in this area for the coming decades. To prevent a long-term care crisis in 2025, action was needed. An important development was the introduction of the concept of the care standard, which describes good care for chronic-care patients based on guidelines and protocols.

The Dutch Diabetes Federation developed the first care standard in 2003. The care standard describes three main aspects of the prevention of and care for chronic diseases: the care, the organization, and the indicators of quality. One other principle of the care standard is the individual care plan, which will be coordinated for and with the patient as well as by a multidisciplinary team of care providers.

The care group was introduced as a new entity to contract, in one market, the different care providers involved in chronic disease management and, in a second, the insurance companies. After the pilot, the contracting of disease management programs for diabetes was nationally covered. One important element of the program is the development of software to not only exchange information between providers, but also manage the treatment plan.

## Results

The Dutch shift to patient-centered care has resulted in real changes in the care delivery system. It has altered the relation between the stakeholders so fundamentally that the existing budgeting system will be replaced completely by 2012. The introduction of health-issue funding for chronic diseases, both for the most common, like diabetes, as well as for rare diseases like cystic fibrosis, has opened new frontiers in healthcare delivery involving the patient and, ultimately, also integrating social care.

The traditional healthcare silos are breaking down. Care providers and patients are looking for state-of-the-art, 2.0 solutions to develop supporting information systems that link to the personal health records of patients to further improve patient quality of life.

## Conclusions

The Dutch approach has created a new dimension in the application of Casemix. It has created direct links between healthcare delivery, costs, and outcomes. The method taken for chronic diseases has linked prevention and healthcare, and it provides a way to extend the paradigm shift of demand-oriented care delivery across the traditional silos.

Another important breakthrough is the creation of a new dimension in Casemix tools. Instead of developing Casemix classification systems primarily based on the statistical analysis of the costs involved in providing care, the new integrated-care approach is based on clinical standards.

So, in the end, the dreams of Codman and Weed will come true. The next generation will be provided with a sustainable healthcare system that involves the patient and uses problem-oriented records, even across institutions.

